# Description of *Deladenus uljinensis* n. sp., and additional DNA barcode data for *Deladenus posteroporus* (Nematoda: Neotylenchidae) from Korea

**DOI:** 10.2478/jofnem-2025-0013

**Published:** 2025-03-29

**Authors:** Abraham Okki Mwamula, Chang-hwan Bae, Yi Seul Kim, Dong Woon Lee

**Affiliations:** Research Institute of Invertebrate Vectors, Kyungpook National University, Sangju 37224, Republic of Korea.; Biodiversity Research Department, Species Diversity Research Division, National Institute of Biological Resources, Incheon, 22689, Republic of Korea.; Department of Entomology, Kyungpook National University, Sangju, 37224, Republic of Korea.

**Keywords:** Molecular characterization, morphology, morphometrics, phylogeny, taxonomy

## Abstract

A new species of the genus *Deladenus* isolated from a dead red pine tree was characterized using morphometric and molecular DNA data. *Deladenus uljinensis* n. sp. is characterized by its lateral fields with six to seven lines, pharyngeal corpus without a distinct median bulb and lacking a chamber, esophageal-intestinal junction located immediately behind the nerve ring, hemizonid located posterior to nerve ring, excretory pore opening within the contour of hemizonid or just at the base of hemizonid, vulva with no lateral vulval flaps, post-uterine sac rudimentary or absent, vulva-anus distance ca. equal to tail length, tail conoid, gradually tapering to a broadly rounded terminus, and slender spicules, 18.5–21.5 *μm* long. The new species was compared with morphologically close species including *D. gilanica, D. brevis, D. pakistanensis*, *D. oryzae, D. uteropinusus, D. aridus,* and *D*. *durus.* Additionally, *D. posteroporus* was also characterized and the population represents the first record of the species outside its type locality. The phylogenetic relationships among species were reconstructed using 18S-rRNA, 28S-rRNA and COI gene sequences. Inferences from the more informative 28S-rRNA gene suggest that *D. uljinensis* n. sp. is a sister species to the morphologically close *D. gilanica.*

## Introduction

The biology and taxonomic position of the tylenchid insect parasites of the genus *Deladenus* (Thorne, 1941) has attracted the interest of many taxonomists. This is especially due to the uncovering of the distinctive insect parasitic phase in several of the known species of *Deladenus* (Bedding, 1968, 1974; Chitambar, 1991; Chizhov & Sturhan, 1998; Siddiqi, 2000). Infective forms are known for at least 14 of the 28 nominal species (Yu et al., 2023). The genus is therefore comprised of two unique groups of species: one comprising species with only mycetophagous forms and another group with a two-phased life cycle (mycetophagous phase and the infective insect-parasitic forms). The genus was created by Thorne (1941) to accommodate those nematodes not possessing a valvular median esophageal bulb. It was differentiated from other genera by the position of the esophageal-intestinal junction, being located immediately behind the nerve ring, a median esophageal chamber present or absent, absence of a post-uterine sac and the V ratio greater than 90% (Thorne, 1941). Some of these characters have since been emended to accommodate related new species, but with wider morphological diversity, including the presence of post-uterine sac and more anterior position of vulva in some species such as *D. megacondylus* (Mulvey, 1969; Sumenkova, 1975).

The modern species-identification approach of integrated taxonomy that combines morphometric data and molecular DNA barcodes has proved to be useful for accurate species identification and delimitation within the group. Recently, several *Deladenus* species have been characterized, and the taxonomic position of the morphologically similar *Contortylenchus genitalicola* (Kosaka & Ogura, 1993) has also been resolved using integrated taxonomy (Heydari et al., 2020; Bonab et al., 2023; Kanzaki et al., 2023). During a nematological survey conducted in 2024 in natural pine forest ecosystems in Korea, *Deladenus posteroporus* (Yu, Gu, Ye, Li, & He, 2017), and an undescribed *Deladenus* population that belongs to the mycetophagous group were recovered from the wood of dead red pine (*Pinus densiflora* for. *erecta*). The new species, designated as *Deladenus uljinensis* n. sp., is herein described based on both morphological and molecular phylogenetic studies. Additional morphological and molecular DNA data of *Deladenus posteroporus* is also supplied. With the exception of *D. valveus* (Yu, Popovic & Gu, 2014) that was isolated from packaging wood intercepted in Ningbo, China, but originating from Korea, these two species represent the first record of the genus in Korea.

## Materials and Methods

### Nematode populations and extraction

The nematode populations were recovered from the sapwood layers of a dead (wildfire-induced mortality) red pine (*Pinus densiflora* for. *erecta*) tree sampled from the Geumgang pine forest in Uljin, Gyeongsangbuk-do Province, Republic of Korea. The wood had signs of insect infestations in the form of tunnel engravings of *Dendroctonus*. Adult males and females of *Dendroctonus* sp. were also collected from the tunnels. However, despite the attempts to recover infective forms, all the 60 collected insects were devoid of *Deladenus* nematode infestation. Nematodes were extracted from the wood cuttings using the Baermann funnel method (Baermann, 1917). Nematode specimens belonging to *Deladenus* were handpicked from the nematode suspension under a Nikon SMZ 1000 stereomicroscope (Nikon). Specimens were subsequently characterized to species level using morphometric and molecular DNA barcode data.

### Morphological characterization

Fresh nematode specimens were heat-killed, fixed in formaline-glycerine proportion, and processed to pure glycerin according to Seinhorst (1959) as modified by De Grisse (1969). The processed specimens were mounted in dehydrated glycerin on permanent slides and examined under a fluorescence microscope. Morphometric data and photomicrographs were taken using a Zeiss imager Z2 microscope (Carl Zeiss) fitted with Axio-vision, a material science software for research and engineering (Carl Zeiss). Line drawings were made under a drawing tube and redrawn using CorelDRAW® software version 24. Species delineation and diagnosis was done following the diagnostic species compendia presented by Bedding (1974); Chitambar (1991); and Aliramaji et al. (2024).

### Molecular characterization

Genomic DNA was extracted from heat-relaxed morphometrically confirmed single female (2 specimens), and single male specimen of *Deladenus* using the DNA extraction kit WizPure™ according to Iwahori et al. (2000). Morphological variations in females were considered by extracting DNA from specimens with and without rudimentary post-vulval sac. Three gene fragments – i.e., nearly full-length 18S-rRNA gene, the D2–D3 expansion segment of 28S-rRNA gene, and the partial COI gene – were amplified and sequenced in this study. The 18S-rRNA gene was amplified as two partially overlapping fragments using two primer sets: 988F (5′-CTCAAAGATTAAGCCATGC-3′) and 1912R (5′-TTTACGGTCAGAACTAGGG-3′), 1813F (5′-CTGCGTGAGAGGTGAAAT-3′) and 2646R (5′-GCTACCTTGTTACGACTTTT-3′) (Holterman et al., 2006). D2A (5′-ACAAGTACCGTGAGGGAAAGTTG-3′) and D3B (5′-TCGGAAGGAACCAGCTACTA-3′) (Nunn, 1992) primer set was used in the amplification of the D2–D3 expansion segment of 28S-rRNA gene, and the partial COI gene was amplified using the primer set COIF1 (5′-CCTACTATGATTGGTGGTTTTGGTAATTG-3′) and COIR2 (5′-GTAGCAGCAGTAAAATAAGCACG-3′) (Kanzaki and Futai, 2002). Polymerase chain reaction (PCR) was performed with a PCR cycler (T100™, Bio-Rad). The thermal cycles for the primer sets D2A/D3B, 988F/1912R, 1813F/2646R, and COIF1/COIR2 were as described by Mwamula et al. (2023). The PCR products were subsequently purified using QIAquick PCR Purification Kit (Qiagen) and quantified using a quickdrop spectrophotometer (Molecular Devices). The purified products were directly sequenced with the primers specified above at Macrogen Inc. The new sequences were edited and submitted to the GenBank database under the accession numbers PQ814252-PQ814256 (for 18S-rRNA); PQ816571-PQ816575 (for 28S-rRNA); and PQ814257-PQ814261 (for COI gene).

### Phylogenetic analysis

By using the BLAST homology search program, the new sequences (18S-rRNA, 28S-rRNA, and COI gene) were compared with those of related species of *Deladenus*, and other comparable sequences of related nematode genera published in GenBank (Yu et al., 2017; Heydari et al., 2020; Jalalinasab et al., 2020; Bonab et al., 2023; Kanzaki et al., 2023; Yu et al., 2023; Aliramaji et al., 2024). Multiple alignments for the three genes were built using ClustalX (Thompson et al., 1997). The sequences of *Acrobeloides varius* (Kim, Kim, & Park, 2017) (MK636580) and *Cephalobus cubaensis* (Steiner, 1935) (AF202161) were used as the outgroup taxa for the 18S-rRNA gene; *Oscheius myriophilus* (Poinar, 1986; Sudhaus, 2011) AY602176, and *Poikilolaimus oxycercus* (de Man, 1895) (DQ059059) were the outgroup taxa for 28S-rRNA gene; and *Ektaphelenchus joyceae* (Kaisa, Harman & Harman, 1995) (MT712703) and *Devibursaphelenchus eproctatus* (Sriwati, Kanzaki, Phan & Futai, 2008) (JN122013) were selected as the outgroup taxa for the COI gene. Bayesian inference (BI) of the phylogenies based on sequences of the three genes was performed using MrBayes 3.2.7 (Ronquist et al., 2012), with GTR + I + G model selected for all datasets. Bayesian inference analysis was run with four chains for 1 × 10^6^ generations, and the Markov chains were sampled at intervals of 100 generations. Consensus trees were generated with the 50% majority rule and the generated trees were edited using FigTree v1.4.4 software. Posterior probabilities exceeding 50% are given on appropriate clades. Intraspecific and interspecific sequence variation was analyzed using PAUP* v4.0a169 (Swofford, 2003).

## Results

**Figure 1:**
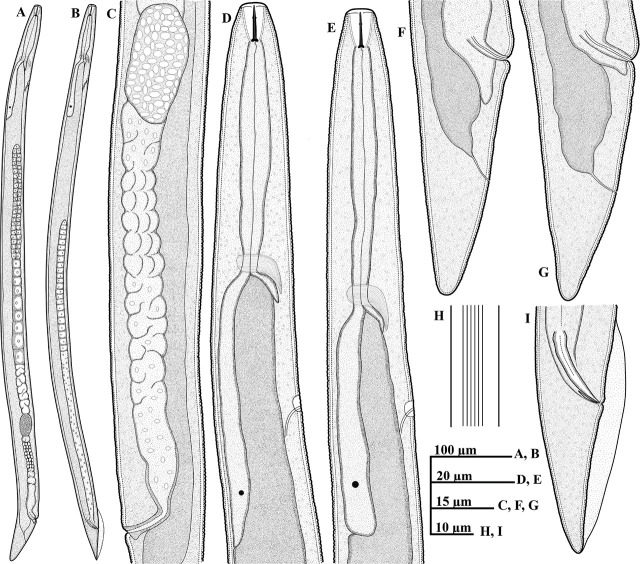
Line drawings of *Deladenus uljinensis* n. sp. (A–I): A: Female whole body; B: Male whole body; C: Female reproductive system; D: Female anterior region; E: Male anterior region; F, G: Female tail region; H: Lateral field; I: Male tail region.

**Figure 2:**
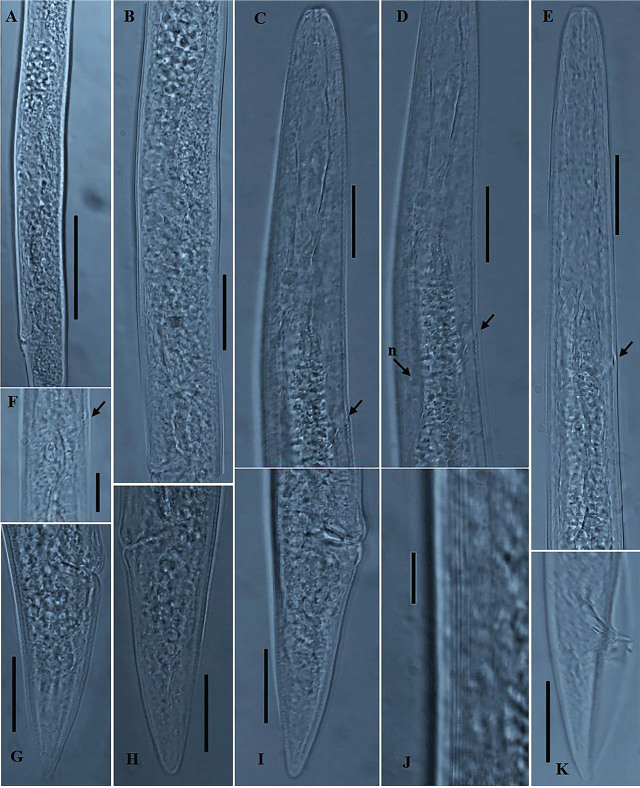
Photomicrographs of *Deladenus uljinensis* n. sp. (A–K). A, B: Female reproductive system; C: Female anterior region; D: Female pharyngeal and glandular region; E: Male anterior region; F: Excretory pore / hemizonid position; G–I: Variation in female tail region; J: Lateral field; K: Male tail region. The arrow labeled n indicates the position of glandular nucleus. All other arrows indicate the collective position of both the excretory pore and the hemizonid (Scale bars: A = 50 *μm*; B, C, D, E, G, H, I and K = 20 *μm*; F and J = 10 *μm*).

*Deladenus uljinensis* n. sp. (Figs. 1 & 2).

### Measurements

See Table 1.

**Table 1:** Morphometrics of mycetophagous population of *Deladenus uljinensis* n. sp. and *Deladenus posteroporus* from Korea.

**Character**	***Deladenus uljinensis* n. sp.**			***Deladenus posteroporus***	

	**Holotype** ♀	♀♀	♂♂	♀♀	♂♂

n	–	30	15	18	10
L	681.0	641.6±53.1 (504.0–736.0)	524.2±24.9 (482.0–561.0)	1007.7±156.3 (819.0–1199.0)	1039.8±62.3 (943.0–1150.0)
a	26.2	24.1±1.9 (21.1–28.3)	24.9±2.4 (21.3–29.3)	45.4±4.9 (36.2–52.6)	48.8±3.0 (42.3–51.7)
b	10.1	9.3±0.7 (7.7–11.0)	8.0±0.9 (6.7–9.3)	11.2±1.4 (9.4–13.7)	11.1±0.7 (10.1–11.9)
b′	4.7	4.5±0.4 (3.7–5.2)	3.9±0.4 (3.5–4.6)	5.4±0.4 (4.6–6.0)	5.6±0.3 (5.0–6.0)
c	21.0	20.8±1.7 (17.5–24.3)	17.8±1.6 (14.8–19.9)	36.2±6.5 (30.0–49.1)	25.5±1.5 (23.4–27.2)
c′	1.9	2.0±0.2 (1.7–2.4)	2.3±0.3 (2.0–3.2)	2.2±0.3 (1.7–2.8)	2.9±0.3 (2.6–3.4)
V	89.9	90.4±0.7 (88.6–91.7)	–	93.0±1.2 (90.9–94.3)	–
G_1_/T	67.0	60.6±5.5 (52.6–71.5)	58.4±3.3 (53.0–66.4)	60.1±8.2 (51.3–74.3)	55.0±5.1 (44.8–58.6)
Lip diam.	6.0	6.6±0.5 (5.0–7.0)	5.6±0.3 (5.0–6.5)	6.3±0.5 (5.0–7.0)	6.0±0.3 (5.5–6.5)
Stylet length	8.5	8.8±0.3 (8.0–9.5)	8.5±0.3 (8.0–9.0)	9.6±0.4 (8.5–10.0)	9.6±0.3 (9.0–10.0)
Anterior to nerve ring	60.0	62.3±4.8 (55.0–72.0)	58.5±5.8 (50.0–70.5)	79.2±8.4 (66.5–99.0)	82.5±6.5 (71.0–90.0)
Oesophageal-intestinal junction	67.0	69.2±5.3 (60.5–80.5)	65.9±6.4 (56.0–78.0)	89.7±7.9 (77.5–109.5)	93.7±8.1 (82.0–105.5)
Anterior to Hemizonid	100.5	99.0±5.5 (90.0–109.5)	89.0±3.9 (82.5–95.5)	110.5±7.9 (97.0–127.5)	111.0±6.0 (99.5–118.5)
Anterior to excretory pore	102.0	102.0±5.5 (91.0–111.0)	91.3±4.2 (83.0–98.0)	111.6±8.2 (98.0–132.0)	112.5±6.9 (102.0–126.0)
Glandular overlap	76.0	72.4±7.0 (57.0–84.0)	69.2±8.4 (52.0–82.5)	97.5±20.9 (70.0–122.0)	91.8±14.0 (80.0–109.5)
Maximum body diam.	26.0	26.7±2.8 (21.5–33.0)	21.2±1.4 (18.5–23.0)	22.4±3.7 (16.5–30.0)	21.4±1.8 (18.5–25.0)
Vulval body diam.	24.5	22.3±2.2 (16.0–26.5)	–	19.5±4.4 (13.0–26.0)	–
Vulva to anus distance (VA)	33.5	29.4±3.4 (21.5–37.0)	–	39.9±4.1 (34.0–49.0)	–
Anal / cloacal body diam.	17.0	15.6±1.4 (12.5–18.5)	12.8±0.8 (11.0–14.0)	12.6±1.3 (10.5–15.0)	14.0±0.4 (13.5–15.0)
Tail length	32.5	30.9±2.2 (26.0–36.0)	29.7±3.1 (24.5–35.0)	28.2±4.1 (22.5–35.0)	40.9±3.6 (36.5–46.5)
Ratio of VA to tail length	1.0	1.0±0.1 (0.7–1.2)	–	1.5±0.3 (1.0–2.0)	–
Spicules	–	–	19.4±1.0 (18.5–21.5)	–	21.9±1.1 (20.0–23.0)
Gubernaculum	–	–	5.6±0.4 (5.0–6.0)	–	6.1±0.7 (5.0–7.0)

### Description

#### Mycetophagous female (n = 31)

Body habitus generally short, stout and straight or slightly arcuate when heat-killed and fixed. Body gradually narrowing toward posterior end post vulval region. Cuticle with fine transverse striae, annulations less than a *μm* apart. Lateral fields with six to seven lines at mid-body, reduced to four lines anteriorly. Lip region low, 1–2 *μm* high, continuous with body contour, anteriorly flattened to slightly rounded. Stylet small, with small posteriorly sloping knobs. Dorsal gland orifice located 1–2 *μm* posterior to stylet knobs. Pharyngeal corpus without a distinct median bulb and lacking a chamber, nerve ring encircling the corpus at 55.0–72.0 *μm* or 39–51% of the total pharynx length (including glandular overlap) from anterior end. Esophageal-intestinal junction located immediately behind the nerve ring. Hemizonid prominent, 4.0–5.0 *μm* long, located 27.0–48.0 *μm* posterior to nerve ring. Excretory pore opening within the contour of hemizonid or just at the base of hemizonid. Glandular overlap long, overlapping intestine by 57.0–84.0 *μm*, with one prominent nucleus visible in the posterior region. Digestive system simple. Reproductive system monodelphic-prodelphic, comprised of anteriorly outstretched ovary, with maturing oocytes in one to two rows, tubular oviduct, axial large oblong-shaped spermatheca with distinct spheroid sperm, crustaformeria with more than four cells in each of the rows, uterus and vagina with moderately sclerotized wall, vulva a wide transverse slit, with moderately protuberant lips, and with no lateral vulval flaps. Post-uterine sac rudimentary (Figs. 1F and 2H), 9.0–11.5 *μm* long, absent in several specimens (mostly in young females). Body gradually tapering post vulva. Vulva-anus distance ca. equal to tail length. Tail conoid, gradually tapering from both sides to a broadly rounded terminus.

#### Mycetophagous male (n = 15)

Generally abundant and functional as evidenced by sperm-filled spermatheca in females. General morphology similar to that of female except for sexual characteristics and a more conical tail. Body generally straight when heat-killed and fixed, thinner and shorter than that of mycetophagous females. Lateral field narrower but similar to that in females. Lip region low, 1–2 *μm* high, continuous with body contour, anteriorly flattened to slightly rounded. The stylet and esophageal region appearing significantly reduced. Hemizonid prominent, located 21.5–38.5 *μm* posterior to nerve ring. Excretory pore opening within the hemizonid or just at the base of hemizonid. Testis outstretched, occupying 53–66% of the total body length. Spicules slender and tylenchoid, gubernaculum thin and short, 5.0–6.0 *μm* long. Bursa well developed, peloderan. Tail longer than cloacal body diameter, narrowing to a rounded terminus.

#### Infective female

Despite the repeated efforts to find infective females and (or) adult parasitic forms from the *Dendroctonus* specimens recovered from the wood tunnels, no infective females or adult parasitic forms were found.

### Diagnosis and relationships

*Deladenus uljinensis* n. sp. is characterized by its short and straight body, lateral fields with six to seven lines at mid-body, pharyngeal corpus without a distinct median bulb and lacking a chamber, esophageal-intestinal junction located immediately behind the nerve ring, hemizonid prominent, located 27.0–48.0 *μm* posterior to nerve ring, excretory pore opening within the contour of hemizonid or just at the base of hemizonid, a large oblong-shaped spermatheca with distinct spheroid sperm, vulva with moderately protuberant lips, and with no lateral vulval flaps, post-uterine sac rudimentary or absent, vulva-anus distance ca. equal to tail length, conoid tail gradually tapering to a broadly rounded terminus, spicules slender and tylenchoid, 18.5–21.5 *μm* long, gubernaculum thin and short, 5.0–6.0 *μm* long.

By having a relatively shorter body and the position of excretory pore in relation to hemizonid, *Deladenus uljinensis* n. sp. is comparable to seven species of the genus, namely *D. gilanica* (Jalalinasab, Esmaeili, Ye & Heydari, 2020); *D. brevis* (Heydari, Abolafia & Pedram, 2020); *D. pakistanensis* (Shahina & Maqbool, 1992); *D. oryzae* (Bajaj, 2015); *D. uteropinusus* (Bajaj, 2015); *D. aridus* (Andrássy, 1957); and *D*. *durus* (Cobb, 1922; Thorne, 1941). It differs from *D. gilanica* by the relatively longer body (504–736 *μm vs* 314–422 *μm*), vulva-anus distance ca. equal to tail length vs. less than tail length, tail gradually tapering to a broadly rounded terminus vs. gradually tapering to a pointed tip, long glandular overlap vs. short glandular overlap (57.0–84.0 *μm* vs. 11–33 *μm*), longer distance from anterior end to hemizonid/excretory pore (90–111 *μm* vs. 61–76 *μm*), a more posterior vulval position (V = 88.6–91.7 vs. 85.4–87.4), and higher c and lower c′ ratios (c = 17.5–24.3 and c′ = 1.7–2.4 vs. c′ = 10.8–14.6 and c′ = 2.4–3.8, respectively); from *D. brevis* by the relatively longer body (504–736 *μm* vs. 367–454 *μm*), longer stylet (8.0–9.5 μm vs. 6–7 μm), hemizonid and excretory pore located posterior to nerve ring vs. hemizonid and excretory pore at the level with nerve ring, vulva lips with no lateral vulval flaps vs. with lateral vulval flaps, lower c′ ratio (1.7–2.4 vs. 2.4–3.2), and longer spicules (18.5–21.5 *μm* vs. 11.3–14.5 *μm*); from *D. pakistanensis* by the stout body (a = 21.1–28.3 vs. 33.0–35.0), its body not abruptly narrowing at posterior of vulva vs. body abruptly or sharply narrowing posterior to vulva, lateral field with 6–7 lines vs. 10–12 lines, excretory pore opening within the contour of hemizonid or at the base of hemizonid vs. excretory pore opening posterior to hemizonid, and vulva lips with no lateral vulval flaps vs. with lateral vulval flaps; from *D. oryzae* by more anterior vulval position (V= 88.6–91.7 vs. 92.6–93.5), lip region not marked off from rest of body vs. marked off from rest of body, excretory pore opening within the contour of hemizonid or at the base of hemizonid vs. excretory pore opening anterior to hemizonid, lateral fields with 6–7 lines vs. indistinct, pharyngeal corpus without a distinct median bulb and lacking a chamber vs. median pharyngeal bulb present, and with median chamber, vulva-anus distance ca. equal to tail length vs. 1.3 times the tail length, and longer spicules (18.5–21.5 μm vs. 16.0–18.0 μm); from *D. uteropinusus* by its shorter and stouter body (504–736 μm vs. 760–820 μm, a = 21.1–28.3 vs. 28.0–36.0), lip region not marked off from rest of body vs. slightly marked off from rest of body, more posterior vulval position (V= 88.6–91.7 vs. 87.2–88.5), pharyngeal corpus without a distinct median bulb and lacking a chamber vs. median pharyngeal bulb present, and with median chamber, lateral fields with 6–7 lines vs. indistinct, vulva-anus distance ca equal to tail length vs. 1.4–1.7 times the tail length; tail conoid gradually tapering to a broadly rounded terminus vs. tail cylindrical, with an inverted U-shaped hyaline region; from *D. aridus* by excretory pore opening within the contour of hemizonid or at the base of hemizonid vs. excretory pore opening posterior to hemizonid; vulva-anus distance ca. equal to tail length vs. almost half the tail length; tail tapering to a broadly rounded terminus vs. acute tip; and from *D*. *durus* by its shorter and stouter body (504–736 μm vs. 820–1360 μm, a = 21.1–28.3 vs. 29.0–50.0), more anterior vulval position (V= 88.6–91.7 vs. 91–93), pharyngeal corpus without a distinct median bulb and lacking a chamber vs. median pharyngeal bulb distinct, and with oval median chamber, excretory pore opening within the contour of hemizonid or at the base of hemizonid vs. excretory pore opening posterior to hemizonid, and tail terminus broadly rounded, vs. narrowly rounded or pointed, with cleft, mucro, or with two or more sharp processes (see Chitambar, 1991).

### Type habitat and locality

The type population was isolated from the wood of a dead red pine (*Pinus densiflora* for. *erecta*) tree stand from the Geumgang pine forest in Uljin, Gyeongsangbuk-do Province. (GPS coordinates: 37° 1′ 12.07″N, 129° 14′ 44.98″E).

### Type material

Holotype female, 20 female, and 8 male paratypes were deposited in the National Institute of Biological Resources of Korea, and 10 female and 7 male paratypes were deposited in the Nematode Collection of Kyungpook National University (KNU), Republic of Korea.

### Etymology

*Deladenus uljinensis* n. sp. was recovered from the Geumgang pine forest in Uljin, Gyeongsangbuk-do Province. Thus, the species epithet *uljinensis* is derived from the locality of its first description, i.e., Uljin.

### *Deladenus posteroporus* Yu, Gu, Ye, Li & He, 2017

(Fig. 3).

**Figure 3:**
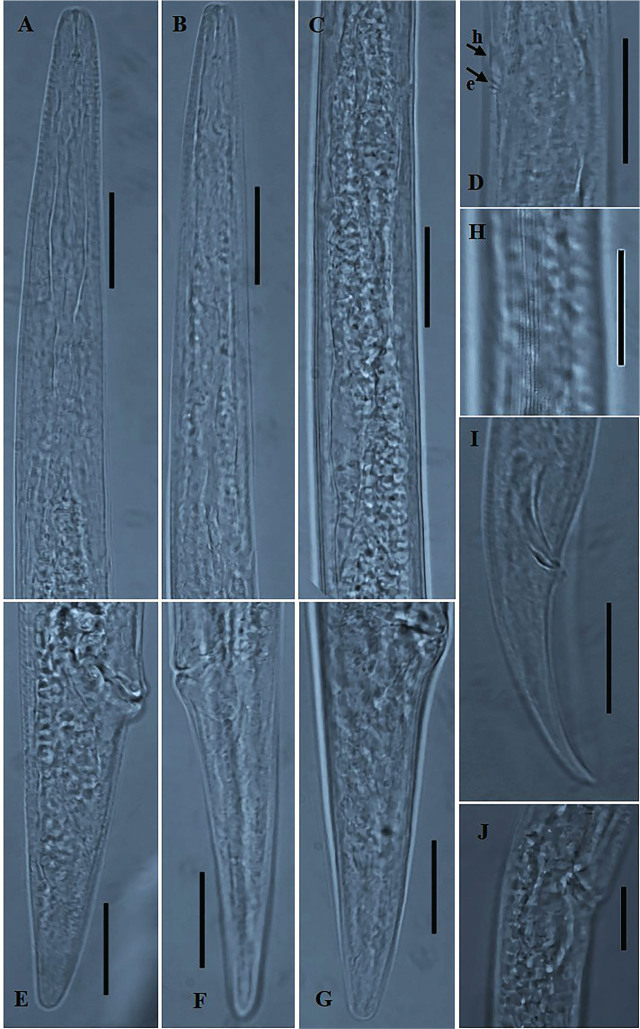
Photomicrographs of *Deladenus posteroporus* (A–J). A: Female anterior region; B: Male anterior region; C: Female pharyngeal and glandular region; D: Excretory pore / hemizonid position; E–G: Variation in female tail region; H: Lateral field; I: Male tail region; J: Vulval region with spermatheca. The two arrows labeled h and e indicate the position of hemizonid and excretory pore, respectively (Scale bars: A–I = 20 *μm*; J = 10 *μm*).

### Measurements

See Table 1.

### Remarks

The morphometrics and general morphology of the studied population agree well with the original species description of Yu et al. (2017), especially the U.S. population with the excretory pore located immediately after the hemizonid, except for some variations in a few characters: for instance, a relatively high c ratio in the current population (30.0–49.1 vs. 26.4–37.1). Also, contrary to the original species description, a short post-vulval sac is present in the mycetophagous females in the studied population (see Fig. 3J). However, although noted as absent in the original description of mycetophagous females (see Yu et al., 2017), their line drawings and photomicrographs indicate the presence of post-vulval sac. Thus, these variations are consistent with the paratype data.

### Type habitat and locality

Similar to *Deladenus uljinensis* n. sp., *D. posteroporus* population was also isolated from the wood of one of the dead red pines (*Pinus densiflora* for. *erecta*) from the same locality (Geumgang pine forest in Uljin, Gyeongsangbuk-do Province; GPS coordinates: 37° 1′ 12.07″N, 129° 14′ 44.98″E).

### Molecular characterization and phylogenetic relationships

The amplification of the nearly full-length 18S-rRNA for the two species yielded fragments of approximately 1700 bp. No intraspecific sequence variation was recorded in the three newly obtained 18S-rRNA gene sequences (PQ814254-PQ814256) of the new species *Deladenus uljinensis* n. sp. In the 18S-rRNA gene phylogeny, *Deladenus uljinensis* n. sp. sequences were grouped in an independent moderately supported subclade in relation to other *Deladenus* species. The two new 18S-rRNA gene sequences of *D. posteroporus* (PQ814252, PQ814253) also showed no intraspecific sequence variation and were also identical to *D. posteroporus* (KY098774) with no sequence variation (0.0%). Sequences of *D. posteroporus* (PQ814252, PQ814253 and KY098774) clustered with closely homologous sequences of *Deladenus proximus* (JF304744 and KF908881), Tylenchomorpha sp. (LC147025 and LC147027), and *Deladenus siricidicola* (AY633447), differing by 9–11 bp (0.6–0.7%), 10–17 bp (0.6–1.0%), and 15 bp (0.9%), respectively.

The D2–D3 region yielded fragments of approximately 750–800 bp. The three D2–D3 sequences of *Deladenus uljinensis* n. sp. (PQ816573–PQ816575) differed by 1 bp, and clustered with *Deladenus gilanica* (MF043927) in a separate moderately supported subclade (pp = 87%), differing from MF043927 by 132–133 bp (19.0–19.4%). The two new D2–D3 sequences of *D. posteroporus* (PQ816571, PQ816572) were identical to the three *D. posteroporus* sequences available in GenBank database with no sequence variation (100% percent identity). In the LSU phylogeny, the five *D. posteroporus* sequences were grouped in an independent well-supported subclade with *Deladenus* sp. (LC801234), *Deladenus* sp. (MG871254), and *D. siricidicola* (MW481657), differing by 44–47 bp (5.7–6.1%), 46 bp (6.2–6.9%) and 48–60 bp (6.7–7.8%), respectively.

**Figure 4:**
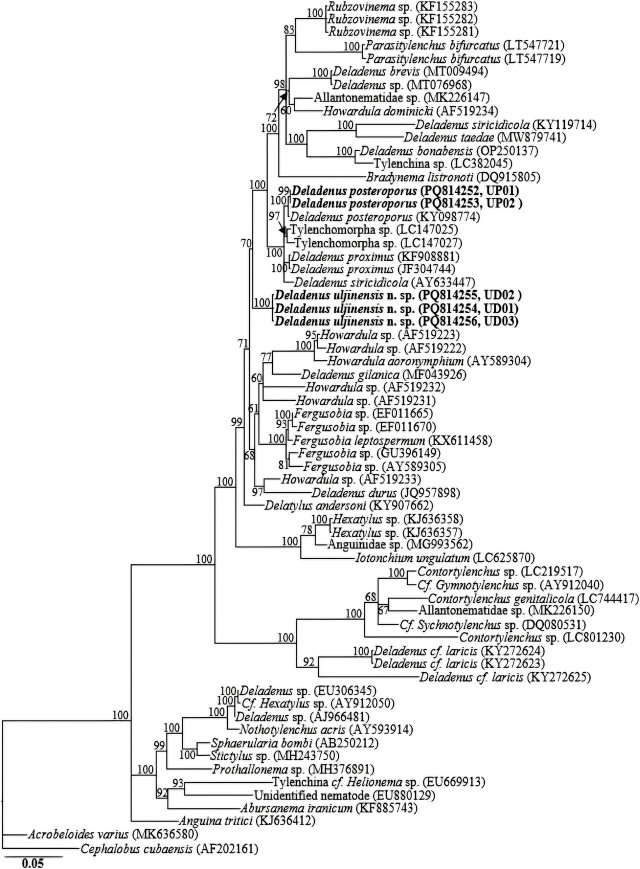
Bayesian tree inferred under the GTR + I + G model from 18S-rRNA gene sequences of *Deladenus* spp. and other related species from various genera. Posterior probability values exceeding 50% are given on appropriate clades. The studied population is indicated in bold text. Outgroup taxa: *Acrobeloides varius* and *Cephalobus cubaensis.*

**Figure 5:**
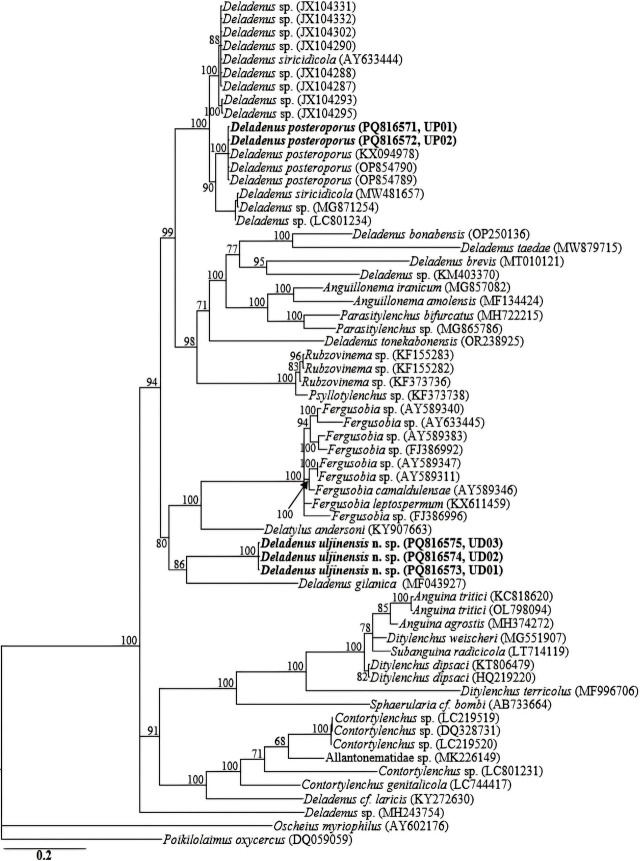
Bayesian tree inferred under the GTR + I + G model from LSU D2–D3 partial sequences of *Deladenus* spp. and other related species from various genera. Posterior probability values exceeding 50% are given on appropriate clades. The studied population is indicated in bold text. Outgroup taxa: *Oscheius myriophilus* and *Poikilolaimus oxycercus*.

**Figure 6:**
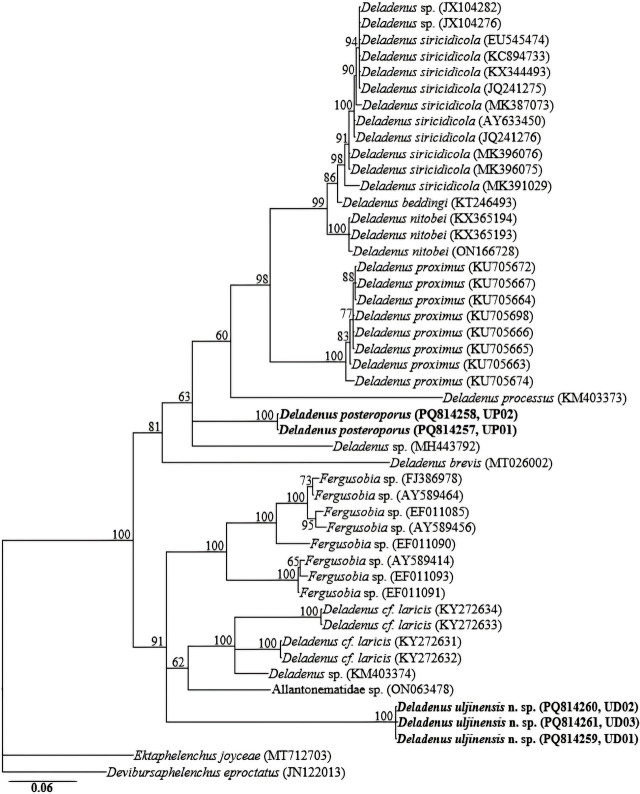
Bayesian tree inferred under the GTR + I + G model from COI gene sequences of *Deladenus* spp. and other related species from various genera. Posterior probability values exceeding 50% are given on appropriate clades. The studied population is indicated in bold text. Outgroup taxa: *Ektaphelenchus joyceae* and *Devibursaphelenchus eproctatus.*

The amplification of the partial COI gene for the two species yielded fragments of approximately 650 bp. Owing to the limited number of COI gene sequences for the genus in GenBank database, the generated sequences for the two species (*Deladenus uljinensis* n. sp.: PQ814259–PQ814261 and *D. posteroporus*: PQ814257, PQ814258) showed relative homology with COI gene sequences of *Fergusobia* spp. *Deladenus proximus* and other unidentified species deposited in GenBank database as Allantonematidae spp.; all with percent identities of 80–85% based on the BLAST homology search program. The sequences of *Deladenus uljinensis* n. sp. (PQ814259–PQ814261) showed a genetic variation of 1 bp., and those of *D. posteroporus*: (PQ814257, PQ814258) were identical with no recorded sequence variation. Reconstruction of the partial COI gene phylogeny grouped each of the two species into independent clades. Sixty-five 18S-rRNA, sixty-six 28S-rRNA and forty-eight COI gene sequences from member species of *Deladenus,* and other related genera, including the newly obtained sequences and outgroup taxa constituted the sequence dataset for phylogenetic analyses. Phylogenetic relationships, as inferred from Bayesian analysis of the dataset with GTR + I + G substitution model, are shown in Figs. 4, 5, and 6.

## Discussion

All the three reconstructed phylogenies (18S-rRNA, D2–D3 expansion of 28S-rRNA and COI gene) suggest that *Deladenus uljinensis* n. sp. is genetically distinct from the available *Deladenus* gene sequences as represented by the Bayesian trees. Inferences from the more informative 28S-rRNA gene phylogeny suggest that *Deladenus uljinensis* n. sp. is a sister species to the morphologically close *D. gilanica.* All the three phylogenies also show that *Deladenus* might be a polyphyletic taxon. Based on the 28S-rRNA gene phylogeny, a number of species, such as *D. bonabensis, D. taedae, D. brevis,* and *D. tonekabonensis* were grouped with member species of other related genera. Similarly, in the18S-rRNA gene phylogeny, *D. durus* and *D. gilanica* were grouped together with sequences of *Howardula* spp. and *Fergusobia* spp. These findings agree with previous studies (Heydari et al., 2020; Bonab et al., 2023). However, it should be noted that these conclusions are based on a limited number of sequences available in the NCBI GenBank for the genus. Thus, accurate resolution of the taxonomic positions of the various species of the genus requires further molecular characterization of the species of the genus and other related groups. This will aid the definitive reconstruction of phylogenetic patterns within the group.
